# The Need for International Terminology and Definitions for Texture-Modified Foods and Thickened Liquids Used in Dysphagia Management: Foundations of a Global Initiative

**DOI:** 10.1007/s40141-013-0024-z

**Published:** 2013-08-24

**Authors:** Julie A. Y. Cichero, Catriona Steele, Janice Duivestein, Pere Clavé, Jianshe Chen, Jun Kayashita, Roberto Dantas, Caroline Lecko, Renee Speyer, Peter Lam, Joseph Murray

**Affiliations:** 1 School of Pharmacy, The University of Queensland, 20 Cornwall St, Brisbane, QLD 4102 Australia; 2 Toronto Rehabilitation Institute, Toronto, ON Canada; 3 Sunny Hill Health Centre, Vancouver, BC Canada; 4 Unitat d’Exploracions Funcionals Digestives, Department of Surgery, Hospital de Mataró, Mataró, Barcelona Spain; 5 Food Science, University of Leeds, West Yorkshire, UK; 6 Department of Health Sciences, Prefectural University of Hiroshima, Hiroshima, Japan; 7 Departmento de Clinica Medica, Faculdade de Medecina de Ribeirao Preto, Universidade de Sao Paulo, São Paulo, Brazil; 8 National Patient Safety Agency, London, UK; 9 Speech Pathology Discipline, James Cook University, Townsville, QLD Australia; 10 Peter Lam Consulting, Vancouver, BC Canada; 11 Ann Arbor Veterans Affairs, Ann Arbor, MI USA

**Keywords:** Dysphagia, Deglutition, Deglutition disorders, Thickened liquids, Texture-modified food, Diet

## Abstract

Conservative estimates suggest that dysphagia (difficulty swallowing) affects approximately 8 % of the world’s population. Dysphagia is associated with malnutrition, dehydration, chest infection and potentially death. While promising treatments are being developed to improve function, the modification of food texture and liquid thickness has become a cornerstone of dysphagia management. Foods are chopped, mashed or puréed to compensate for chewing difficulties or fatigue, improve swallowing safety and avoid asphyxiation. Liquids are typically thickened to slow their speed of transit through the oral and pharyngeal phases of swallowing, to avoid aspiration of material into the airway and improve transit to the esophagus. Food texture and liquid modification for dysphagia management occurs throughout the world. However, the names, the number of levels of modification and characteristics vary within and across countries. Multiple labels increase the risk to patient safety. National standardization of terminology and definitions has been promoted as a means to improve patient safety and inter-professional communication. This article documents the need for *international* standardized terminology and definitions for texture-modified foods and liquids for individuals with dysphagia. Furthermore, it documents the research plan and foundations of a global initiative dedicated to this purpose.

## Introduction

Each year, individuals of all ages all around the world are diagnosed with feeding or swallowing difficulties (dysphagia). At its broadest, dysphagia can be described as difficulty moving food, liquid, saliva or medication from the mouth to the stomach. Over the last 30 years, as the field of dysphagia research has grown, more technical definitions delineating the difference between oropharyngeal and esophageal dysphagia have emerged [1]. Oropharyngeal dysphagia is specifically classified by the World Health Organization in the International Statistical Classification of Diseases and Related Health Problems ICD-9 and ICD-10 (787.2, R13). The consequences of oropharyngeal dysphagia include dehydration, malnutrition, aspiration and asphyxiation and a negative impact on quality of life and social participation in eating and drinking. Conservative estimates suggest that 8 % of the worldwide population experiences difficulty eating regular food and drinking regular fluids because of dysphagia. According to the Population Reference Bureau this amounts to a sobering 99 million individuals from the developed world. Although dysphagia touches individuals across their life span, it is most insidious at either end of life, with infants and the elderly being frequently affected. Although sufferers are sometimes unaware of the disorder, oropharyngeal dysphagia is a highly prevalent clinical condition as it affects more than 30 % of patients with stroke [2], 60–80 % of patients with neurodegenerative diseases [3], 10–30 % of adults aged 65 and older [4] and more than 51 % of institutionalized elderly patients [5]. Treatment to rehabilitate swallowing function and compensation to ‘work around’ swallowing difficulties are common pathways for managing dysphagia. Although promising treatment research is being pursued, the provision of texture-modified foods (e.g., purée) and thickened liquids have become a cornerstone of dysphagia management.

Today’s world looks very different from what it was even 30 years ago. Technology and travel have reduced distances and re-created a very large ‘village’: a global village. With the movement of both patients and health professionals around the world, the use of globally recognized terms for foods and liquids has clear advantages for facilitating the delivery of safe and quality therapeutic products to individuals with dysphagia. Further, in order for researchers to accurately determine which texture-modified diets or thickened fluids provide the greatest therapeutic benefit to our patients, it is critical that we all use the same terms, definitions and measurable characteristics. Standardized terminology and definitions will allow for consistent communication among health professionals, care providers, researchers and industry partners to improve quality of care and safety for patients. Wouldn’t it be wonderful if “nectar thick” liquid or “minced” texture could mean the same thing in all parts of the world? This article aims to expose the need for international standardized terminology, document current national standardized terminologies and describe an international initiative for standardization of texture-modified foods and liquids as used in dysphagia management across the lifespan.

### Why International Standardization?

Two primary reasons for pursuing international standardized terminology are: (1) improved patient safety and (2) evolution of the field of dysphagia to deliver better treatment outcomes. With regard to patient safety, texture-modified foods are generally provided to reduce risks associated with choking, while thickened liquids are provided to reduce risks associated with aspiration. Consequently, the ingestion of texture-modified foods and thickened fluids is rarely the diet of choice, but rather a diet of necessity for the person with dysphagia if they wish to maintain safe oral intake of nutrients [6].

### Texture-Modified Food

It is easy to take the process of chewing and swallowing food for granted. The skill required to break down solid food, mix it with saliva, collect it into a cohesive bolus and transport it to the posterior of the oral cavity for swallowing can be challenging to near impossible for individuals with oropharyngeal dysphagia. Physiological or anatomical changes can increase the effort required in oral preparation so significantly that reduced amounts are consumed, and maintaining nutrition by oral means alone is compromised. For some individuals inefficient chewing can be a choking risk. Choking refers to an inability to breathe because the airway is blocked, constricted or swollen shut. Asphyxiation refers to inadequate delivery, uptake or utilization of oxygen by the body’s cells, and it is often accompanied by carbon dioxide retention. When a bolus becomes lodged in the larynx or pharynx such that the airway is compromised, choking or asphyxiation may occur. Both terms are used in the literature to capture this phenomenon. The risk of death from asphyxiation in the general population has been reported as 0.66 fatalities per 100,000 people [7]. The risk of asphyxiation increases with cognitive impairment, oral stage swallowing impairment and intellectual disability [7]. Information pertaining to choking hazards for adult and pediatric populations has been gathered from research relating to autopsy results and asphyxiation studies [7]. It would be unethical to design research for individuals with dysphagia where one of the potential outcomes was a fatal choking risk.

Mature individuals with partial or missing dentition are reported to be more prone to asphyxiation from food [8]. In one sample of 120 adults, autopsy reports of asphyxiated foods included: meat, pastries, bread, fruit, vegetables, egg and cheese. Hard-textured and fibrous foods require sufficient chewing strength and stamina to ensure the food is broken down sufficiently to avoid being a choking risk. Individuals with swallowing difficulties have been shown to have significantly reduced bite force when compared with those without dysphagia [9]. Reduced bite strength may be a function of aging associated with weakened musculature or the result of deterioration in health. Bite force is also associated with the number of remaining teeth, with a correlation between increasing prevalence of dysphagia apparent in those with fewer than 13 remaining teeth. It is also important to appreciate that bite strength varies as a function of gender and ethnicity. For example, males generally have a higher bite force that females, and ethnic Eskimos have a higher bite force than white Americans [10]. In addition, there is evidence of wide inter-individual variation (110–370 N force), helping to explain why consumers may perceive the same food differently [10]. Those with high bite-force capabilities may perceive a food as weak or fragile, while other consumers with lower bite strength may struggle to fracture the same food substance.

In a retrospective study of 44 autopsy files for food asphyxia, 61 % of the deceased were described as edentulous or having a significant number of missing teeth [11]. Individuals who wear dentures have a different oral experience than those with teeth. Intact teeth are sensitive to vibration, in addition to force and pressure [10]. This tactile sensation allows us to appreciate crisp and crunchy food. However, a loss in this specific tactile sensation impacts on the perception of the force, and pressure required for chewing. Such is the case for individuals who wear dentures. In a small study (*n* = 15) that looked at the impact of dentures on chewing, swallowing and choking, Berretin-Felix et al. [12•] found that more than 80 % had chewing difficulties and 40 % reported swallowing difficulties. Of interest, 33 % reported difficulty with ingesting liquids, 13 % reported coughing and 46 % reported choking. The participants were then fitted with five dental implants in the mandibular arch, with their dentures converted to an implant-supported prosthesis. The dental implants integrate with the bone in the lower jaw to provide permanent fixation and stability. The participants were tracked at 3, 6 and 18 months post-implant surgery. From 3 months post-surgery statistically significant declines in reported rates of masticatory difficulties, swallowing difficulties, difficulty ingesting liquids, choking and coughing were recorded. These results suggest that individuals who wear dentures are at higher risk for safe management of solids and liquids.

Although it has been established that improperly chewed food is a choking risk, a lack of dentition and/or chewing ability impacts nutritional adequacy. In a study of over 50,000 participants, Joshipura et al. [13] found that edentulous men consumed fewer nutritious fibrous foods and more saturated fats and cholesterol than men with 25 or more teeth. Furthermore, Ansai et al. [14] found that the mortality rate increased by 2 % for each missing tooth in individuals over 80 years, studied over a 4-year period. In particular, difficulty chewing *soft* food was associated with an increased risk of mortality (hazard ratio 2.38). These results held even after adjustment for gender, physical health and smoking status.

In order to limit the risk of choking, solid texture foods are often altered such that they require little or no chewing. Foods that require less chewing, and that are cohesive and moist, are typically seen as the easiest, and perhaps by these characteristics, safer to swallow. As shown in the discussion below, puréed foods feature heavily in national terminologies of texture-modified foods for dysphagia management. In a detailed review of texture-modified foods and liquids spanning 1981–1996, Penman and Thomson found wide variation in the degree of texture modification used and many different texture descriptions [15]. Texture-modified diets typically ranged from two to five categories of altered food consistency [15, 16]. The most commonly described model of progression is noted in Table 1.

**Table 1:** Model of progression of diets used for dysphagia (adapted from Penman and Thomson) [15]

Food grading	Description of food texture
Liquidized/thin purée	Homogenous consistency that does not hold its shape after serving
Thick purée/soft and smooth	Thickened, homogenous consistency that holds its shape after serving and does not separate into liquid and solid component during swallowing, i.e., cohesive
Finely minced	Soft diet of cohesive, consistent textures requiring some chewing (particle size most often described as 0.5 × 0.5 cm)
Modified normal	Normal foods of varied textures that require chewing, avoiding particulate foods that pose a choking hazard (particle size most often described as 1.5 × 1.5 cm)

Texture modification for infants is a normal response to the gradual development of their chewing skills. In western cultures, infants typically progress from foods requiring no chewing (purée), through foods with soft lumps, to mashed, chopped and soft food textures. There is limited literature available regarding the pediatric dysphagia diet, and any literature-based guidance for this population is drawn from information on normal feeding development. Carruth and Skinner [17] published milestones for infant feeding development. For example: eats food with tiny lumps (mean 8.7 months); chews soft foods (9.4 months); chews firm foods (10.5 months); chews and swallows firm foods without choking (mean 12.1 months); and chews foods that produce juice (15.2 months). The ability to tolerate unmodified foods depends on the infant’s oral experiences and the ability of the lips, tongue and jaw to work as independent and co-dependent units. The ability to chew foods with firmness, hardness and different textures is also affected by the pattern of normal dental eruption in the first 2 years of life [17]. Children do not gain their second molars until they are 20–24 months of age, and they are thus not well equipped for grinding of food particles until that time [18]. Children typically achieve an adult pattern of muscle activation for the oral and pharyngeal stages of swallowing after 4 years of age [19, 20]. To avoid accidental choking, most child development texts and websites recommend delaying the introduction of hard dry foods until after 3 years of age when sufficient biting and chewing skills have developed. Factors associated with risk of asphyxiation of food items in infants and children include: (1) inadequate dentition, (2) difficult food textures, (3) concurrent neuromuscular disease (e.g. cerebral palsy), and (4) need for supervision during eating.

Texture-modified food is usually provided to reduce risks associated with choking while eating. A specialist in dysphagia will determine the person’s ability to safely manage food textures and recommend an optimal diet based on this assessment. Much like a medical prescription, the product should consistently meet certain standards for safety. Provision of an incorrect product can result in adverse events. Two hundred autopsy studies of the elderly found that semi-solid foods contributed to a large number of asphyxiations [8]. Coronial inquests have identified staff confusion regarding food textures and their labels as factors specifically noted to contribute to patient death [21••].

It has been estimated that the prevalence of modified food texture use in long-term care facilities is somewhere between 15 and 30 % [22]. An American task force identified 40 different names used to label solid food, while an Australian study found 95 different labels used to describe texture-modified foods [6]. If such variability exists within countries, global inconsistency is likely to be staggering.

### Thickened Liquids

Safety of swallowing of liquids is another area of concern for individuals with dysphagia. As opposed to the concerns around asphyxiation and choking, professional anxiety around swallowing liquids relates to the risk of aspiration and the sequelae of life-threatening chest infection. Regular (thin) fluids require finely tuned coordination and timing between a multitude of muscles and nerves to allow the bolus to flow through the pharynx and past the entry to the airway on its way to the esophagus and stomach. Thick liquids tend to flow more slowly, and there is an assumption that slower flow allows better control during swallowing. Safety of swallowing liquids is often evaluated during a clinical examination. Further formal evaluation may follow using instrumental techniques such as videofluoroscopy (modified barium swallow) or fiberoptic endoscopic evaluation of swallowing (FEES). These investigations may also help determine whether the individual will benefit from thickened liquids and if so the thickness level.

The literature shows that the use of thickened fluids for dysphagia has undergone an exponential increase since the beginning of the century, with early studies dating from 1993. Thick fluids have been credited with inducing a number of physiological changes to swallowing biomechanics that may contribute to safer swallowing. A 2013 study using a 3D CT scanning system clearly documented differences between the physiological response of healthy individuals to swallowing thin and thickened liquids [23]. Specifically, thin liquids were found to arrive sooner and remain longer in the hypopharynx than honey-thick liquids. True vocal cord closure was reported to occur sooner and last for longer than for thick liquids [23]. In other research, swallowing thick or dense fluids (1) results in smaller sip sizes [24], (2) elicits a longer period of deglutition apnea and longer bolus transit times [25], (3) improves timelines of swallow reflex initiation relative to the bolus arriving in the pharynx [26] and (4) results in improved airway protection and more normal swallow-respiratory patterns [25, 26]. There is little difference in suprahyoid muscular activity between liquids and thin pastes (i.e. apple sauce and chocolate pudding) reported [27]. However, it is important to be aware that the amplitude of maximum muscular activity has been found to increase from (1) liquids to thick pastes (i.e. cheese spread and peanut butter) and (2) from thin pastes to thick pastes. Total swallow duration increases from liquids to thin pastes to thick pastes. Raut et al. [28] therefore, cautioned that for ‘weak and feeble patients’ or those with pharyngeal phase dysfunction, the ability to swallow thick and viscous substances may be even more *impaired* because of difficulty generating the tongue and pharyngeal pressures required to move the bolus. Consequently, the use of thickened liquids can contribute to incomplete clearance from the pharynx and a higher risk of aspiration from post swallow residue.

Research is limited in relation to the number of different levels of thickened fluids that might be required to meet the different needs of patients with dysphagia. Most studies have compared swallowing performance at either end of the viscosity continuum, for example demonstrating benefits when comparing thin fluids versus paste thickness [26, 27]. Perhaps this is where the perception by swallowing specialists that ‘the thicker the liquid, the safer the swallow’ originates. However, patients who aspirate very thick liquids tend to have worse health outcomes, including fatal ones [29]. Clavé et al. [30] reported that both nectar-thick (274 mPa s) and pudding-thick (3,931.2 mPa s) liquids reduced aspiration in patients with diagnoses of brain injury and neurodegenerative diseases. Reduced aspiration was also reported by Robbins et al. [31] in adults with Parkinson’s disease and/or dementia who aspirated thin liquids and were then randomly assigned to receive either nectar-thick liquids (300 mPa s) and a thicker consistency that was labeled honey-thick, but technically fell in the pudding-thick range (3,000 mPa s).

Since the inception of thickened liquids as a treatment for dysphagia, the Logemann and Robbins et al. [29, 31] studies are the only known randomized control trials (RCT) for dysphagia fluid thickness reported in the literature. In an RCT continuing from the initial study [31], Robbins et al. [31] found that for individuals with dementia in long-term care settings, there was a two-fold increase in the incidence of pneumonia for individuals who consumed ‘honey-thick’ (as noted, actually ‘pudding-thick’) liquids over a 3-month time interval as opposed to individuals receiving ‘nectar-thick’ fluids. These data also show that while the prescription of thickened liquids may reduce aspiration when compared with thin liquids, it does not prevent the development of aspiration pneumonia. Aspiration and aspiration pneumonia are related, but are two distinct outcomes.

Robbins et al.'s [31] use of the label “honey-thick” to describe a liquid of 3,000 mPa s clearly demonstrates a challenge for the dysphagia field with respect to terminology and product classification. When the viscosity of a product does not match the expected range implied by its label, there is potential for confusion and inappropriate use of products for people with dysphagia. In the Robbins et al. [31] study, negative findings of longer periods of hospitalization, poorer health outcomes and more frequent antibiotic use should have been attributed to pudding-thick rather than to honey-thick liquids. Clinicians and researchers need to be confident that the name, definition, and rheological value of ‘nectar-thick’ in North America, for example, is the same as ‘nectar-thick’ in Europe, South America, South Africa, China, New Zealand and indeed the rest of the world.

Penman and Thomson’s [15] review of nomenclature for dysphagia diets demonstrated a large variation in the number of consistencies of thickened fluids offered ranging from a single level of ‘thickened only’ to up to six variations of fluid thickness (see Table 2). Atherton et al. [6] reported 39 different labels for thickened liquids used by clinicians in Australia, used prior to national standardization. In addition, words used to describe thickened liquids in the dysphagia literature are also variable and often not well defined. Researchers have used the words ‘pudding,’ ‘thickened liquid’ and even ‘honey-thick liquid’ without providing a definition of what they mean by these terms [23, 25–28]. If adjustment of fluid thickness has a reliable and demonstrated impact on improved health outcomes, then the exact thickness of the liquids needs to be identified, described and reported in such a way that it can be reliably reproduced.

**Table 2:** Themes of thickened fluid classification based on Penman and Thomson’s review of dysphagia diets [15]

Fluid name and level	Description of fluid thickness
Level 1—nectar	Like nectar
Level 2—honey	Like honey
Level 3—pudding	Like pudding
Thin	Water and all juices thinner than pineapple
Thick	All other liquid including milk and any juice not classified as thin
Thickened	Liquids thickened with starch to puréed consistency
Watery	Water, tea, coffee
Milky	Milk and most fruit juices
Single cream	Ensure Plus and Enterat
Double cream	Tomato juice, thinned puréed fruit, creamed soups
Custard	Cheese or custard sauce, smooth yogurt
Semi-solid	Thick-set yogurt, blancmange, mashed potato

Infants with dysphagia are also provided thickened liquids; however, these are often thinner than those provided to adults in order that they can pass through a nipple/teat. Studies have objectively reported the values of thickened liquids used for infants; however, evidence supporting the therapeutic benefit of specific thickness levels remains elusive [32, 33]. In the review of uptake of Australian standardized terminology and definitions, respondents indicated that the lowest level reported by the current Australian standards (level 150, midly thick), was too thick for infant use [21••]. A thinner consistency and an appropriate descriptive label are required to suit their needs.

### Measuring the Characteristics of Texture-Modified Food and Thickened Liquids

The question of how thickness is measured has been gathering momentum over the last 16 years. Collaboration with experts in rheology and material property characterization is helping to drive the field forward [34–37]. Inter-professional collaboration between health research and engineering is critical to the development, measurement and description of products that aid safe swallowing. Although objective measurement of fluids has been gaining traction over the last decade, formal methods of measuring texture-modified foods that are reliable and accessible are still in their infancy. Determining the features of texture-modified foods that most need measurement for consistency of products and their role in safe swallowing is an emerging field [10].

### Supporting Individuals with Dysphagia: The Role of Industry

Individuals with dysphagia may be discharged from the hospital still requiring texture-modified foods and liquids. Individuals in aged care facilities rely on such products. Industry has supported individuals with dysphagia by developing: (1) powders and liquids that thicken fluids to allow patients choices in their thickened beverages, (2) pre-thickened liquids in a variety of flavors that take the guess work out trying to produce a consistent fluid thickness level and (3) pre-packaged texture-modified food for convenience. There are inherent issues relating to consistent thickness levels when individuals make up their own drinks using thickening agents [35, 38]. A recent study shows that thickened fluids for dysphagia patients prepared in the hospital by clinicians were significantly different from those prepared in the laboratory, yet both used the same instructions [39•]. In addition, little is known regarding whether the ‘nectar-thick’ or ‘purée’ of company A is in fact equivalent to the ‘nectar-thick’ or ‘purée’ of company B. This presents a safety issue. For industry the problem is compounded by differences in national terminology. The product label may not be able to accommodate three or four different terms for products manufactured centrally but distributed internationally. These realities lead to an increase in chances for mistakes to be made.

### Published National Standards

Over the last decade, there has been a move toward national standardization of terminology and definitions for texture-modified foods and liquids. We are aware that Australia, Ireland, Japan, New Zealand, Sweden, the UK, the USA and Denmark have published national descriptors [6, 40–43, 44••, 45, 46]. At the time of writing, Canada is in the process of developing national guidelines. These published materials are summarized in Tables 3 and 4. Unpublished information, provided by colleagues from Spain, The Netherlands and Brazil, are also included in the tables. Advice from colleagues in these countries suggests these are the most common names used, although variation exists from region to region, north to south and east to west.

**Table 3:** International terminology for thickened liquids

^a^Shear rate 50 s^−1^; both cP and mPa s are used in the literature as the unit of viscosity, 1 cP = 1 mPa s

**Table 4:** International terminology for texture-modified food

A review of these tables demonstrates both similarities and differences. In addition to regular food, there are typically three levels of texture-modified foods, with some countries having as many as five. Some countries differentiate food textures based on the size of particles included in the diet. This has the benefit of providing a means of quality control of food service kitchens. The words ‘soft’ and ‘purée’ feature consistently in western dysphagia diets. The Japanese texture-modified food standards blend textural information with nutritional information. Some standards include liquids, such as soups, as part of their ‘food texture modifications.’ However, differentiation between solids and liquids appears to form the basis of separation into thickened liquids versus texture-modified foods for other countries. For liquids where there are only two levels of differentiation, ‘thin’ and ‘thickened’ are the dichotomous labels used.

For countries with multiple levels of thickened liquid differentiation, there are typically three to four levels of increasing thickness. The American and Japanese descriptors include specific rheological measures for their liquids, and both use a shear rate of 50 s^−1^. Numbered gradings or stages of 1–3 are common. The Australian system has used wider variation in the numbering system (level 150, 400 and 900), described in their publication as similar to sunscreen protection factor ratings where increased sun protection is implied from larger numbers (SPF 15, 30) [6]. For liquids, larger numbers indicate increased thickness levels. The Australian system employed a dual numerical and descriptive labeling system. Descriptions of thickness such as mildly thick, moderately thick or extremely thick can be seen in the Australian, New Zealand and Irish standards that share similar origins [6, 40, 42]. The American standards use terms reflective of real fluids that people may be familiar with such as ‘nectar’ or ‘honey’ or indications of thickness by the utensil required to consume it (e.g., spoon) [45]. Note that some countries use colors in addition to descriptors to identify different levels. Colors are widely used in society as an additional means of communication, for example, the universal understanding of the traffic light system.

The Japanese Society of Dysphagia Rehabilitation (JSDR) appears to be the most advanced in providing measurable specifications for both liquids and foods. The JSDR is also in the process of converting rheological measures for liquids expressed in mPa s to line-spread measure values (cm) [41]. The Japanese system of identifying texture-modified foods is very advanced, incorporating energy content (kcal), protein (g) and measures of hardness, adhesiveness and cohesiveness for each food level. Measures are also differentiated on whether the food is served cold (15 °C) or warm (45 °C). With extremely texture-modified foods, Japanese clinicians also determine whether purée or jelly textures are safer or easier to swallow. The Japanese system also includes a recommendation for 30–40 % weight/volume for barium sulfate to be added to foods/liquids to ensure substances are radiopaque for videofluoroscopy examination. Addition rates of locally available thickening agents (gels, powder thickener, agar) per 100 ml liquid barium are also available.

Although national standards have been implemented in a number of countries, to the best of our knowledge only one country has evaluated uptake of their national descriptors. In the review of the uptake of Australian standards, Jukes et al. [21••] reported that over a 3-year period 88 % of respondents had either fully or partially implemented the new terminology. Two key benefits emerged regarding the importance of national standardized terminology: (1) perception of increased patient safety and (2) improved inter-professional collaboration.

### International Initiative

Although it is possible to produce tables of international comparisons such as those included in this article, the universality of a single understood terminology for texture-modified foods and fluids has great appeal. Improved patient safety and inter-professional collaboration, as reported in the Australian study exploring the uptake of national standards, serve to benefit individuals with dysphagia worldwide. Given the success of these initiatives at a national level, a bold idea has emerged to pursue and embrace international standardization.

In June 2012, a group of founding task force members representing many facets of the dysphagia journey including medical, nursing, allied health, patient safety, industry, chemical engineering and food technology expertise came together. The result of this meeting was the formation of the International Dysphagia Diet Standardisation Initiative (IDDSI; web address: www.iddsi.org). IDDSI is an independent and not-for-profit entity. The initiative was officially launched in October 2012 at the meeting of the European Society of Swallowing Disorders in Barcelona, Spain.

The aim of this global initiative is to develop international standardized terminology and definitions for texture-modified foods and thickened liquids for individuals with dysphagia of all ages, in all care settings, and all cultures. Through collaboration the group intends to deliver international standardization of terminology and definitions by 2015.

A 2-year time frame and a four-stage plan have been produced to bring international standardization to fruition. This document marks one of the first official steps in documenting the process. The scope of the project is listed below:

This project will seek to:

- Determine the number of food texture classifications to be defined for international standardized use (inclusive of adult and pediatric requirements).
- Determine the number of fluid thicknesses to be defined for international standardized use (inclusive of adult and pediatric requirements).
- Determine standard names/identifiers for each of the food and fluid thickness classifications. Include objective measurement data and best scientific evidence where it exists paired with low-technology and descriptive measures.
- Identify examples of foods that are appropriate for each of the classifications, including culturally specific foods.
- Gain input and consensus from key stakeholders regarding: the number of classifications, definitions, appropriate foods/fluids and names/identifiers for the food textures and fluid thickness levels to be used.
- Communicate the international standards to key stakeholders and commence education of stakeholders regarding the international standards.

It should be noted that regardless of the number of classifications that are ultimately chosen to describe food and fluid texture modification, there is no a priori intention that all classifications of texture modification will necessarily be used, merely that they are all clearly identified and defined. Future research will be needed to explore and confirm whether physiological differences or therapeutic benefits exist for different levels of texture modification, as delineated by the proposed standards.

This project will not: (1) involve the implementation or ‘roll out’ of the international dysphagia diets once developed, (2) cover the nutritional adequacy of texture-modified diets or fluids, (3) develop guidelines for clinical application and clinical outcomes, (4) address patient acceptability of modified diets or fluids or (5) address issues of the reliability in consistency of thickened fluids during production.

### Project Plan

#### Stage 1—Collaboration and Consolidation (Early to Mid 2013)

A review of existing published national terminology and definitions will be conducted and published. Current accumulated tables will be sent to associations for healthcare professionals involved in dysphagia care for validity and to capture countries that may be in the process of standardization. Four work streams will be established to support the project, these being: communication, scientific, industry and stakeholder work streams.

#### Stage 2—Gathering the Evidence (January 2013–September 2013)

All work streams will work concurrently. The scientific work stream will conduct a multicenter systematic literature review to identify existing evidence for the use of texture modification and to glean definitions of texture modification from publications. The scientific work stream will draw on the expertise of clinical researchers, chemical engineers and food technologists in reviewing and ranking the quality of identified literature and in synthesizing any recommendations or findings that result. The scientific work stream will publish the results of the systematic review relating the evidence for the use of texture-modified foods and liquids and definitions reported in the literature. Concurrently, the communication, industry and stakeholder work streams will collaborate to develop and send out surveys to stakeholders regarding their needs for standardized terminology.

With regards to stakeholders, the authors consider that a key tenet of the project is to keep the ‘person with dysphagia’ firmly at the center. Thus, a consumer or ‘patient’ representative to inform the project is seen as critical. Similarly the perspective of the caregiver should also be recognized. Professional groups from the health industry that should be captured in stakeholder surveys include: allied health (dietitians, speech pathologists, occupational therapists), physicians, nurses, dentists and likely other groups. Other stakeholders include individuals from public health, quality improvement, safety and administration experts. Food service representatives and catering representatives were also identified as providing valuable information regarding the application of the descriptors in practice. For example, can the various descriptors be prepared in large-scale commercial settings, on-site settings and smaller settings? Industry was also identified as having a key role in this initiative with a dedicated work stream to address their input and needs in order to provide the best care to individuals with dysphagia.

#### Stage 3—Interlacing Technical and Research Evidence with Clinical and Cultural Needs (October 2013–January 2014)

All work streams will work concurrently to merge information gathered from the technical and scientific reviews of the evidence with clinical and cultural needs with respect to standardized terminology. The data gathered will allow consolidation and composition of a draft set of international standardized terminology for texture-modified foods and thickened liquids for individuals with dysphagia.

#### Stage 4—Consolidation and Publication (February 2014–January 2015)

From February to June 2014, the work streams will develop further surveys to allow feedback on draft international standards. In the latter half of 2014, they will review feedback provided by stakeholders, allowing fine-tuning of the international standards. At the conclusion of Delphi-style surveys, the initiative will seek endorsement of the international standards from regulatory bodies, professional associations and societies. By January 2015, a manuscript proposing the international standards is planned.

### Challenges and Moving Forward

The authors are strongly of the opinion that persons with dysphagia should be the central focus of the project. A risk of ‘professional silos,’ where professional groups are concerned with ‘ownership of concepts,’ has been identified as a potential challenge. International terminology should be able to span the continuity of care from an acute hospital setting through to the community. The language chosen will need to be sympathetic to this need. In addition, language that can be understood across technical, cultural, professional and non-professional boundaries is regarded as important. The need to address both adult and pediatric needs has been highlighted. A challenge has been identified with respect to whether a global terminology will be practical given diverse cultural and linguistic needs. Surveys and focus groups are the vehicles proposed to answer these challenges.

## Conclusions

The benefits of standardized terminology and definitions have been demonstrated in countries around the world (the UK, USA, Australia, Japan, New Zealand, Ireland). It is our belief that international standardization will result in improved safety, reliability and quality of texture-modified foods and liquids for the vulnerable population of people with dysphagia. For government, industry, hospitals and skilled nursing facilities, standardized terminology promises benefits through the reduction of costs associated with waste and errors. For all parties, there is a desire that the implementation of standardized terminology and definitions would lead to a reduction in critical incidents where death may be an outcome. Patient safety is at the core of this initiative. The benefits of inter-professional collaboration have been ably demonstrated in the development and publication of the British Dysphagia Diet Food Texture Descriptors [44••]. The international initiative looks forward to building on this foundation of inter-professional collaboration for a successful outcome.
